# Digitally measuring solar ultraviolet radiation in outdoor workers: A study protocol for establishing the use of electronic personal dosimeters in Portugal

**DOI:** 10.3389/fpubh.2023.1140903

**Published:** 2023-03-31

**Authors:** Marília Silva Paulo, Cara Symanzik, Melanie R. Maia, Luís Velez Lapão, Fernanda Carvalho, Sven Conneman, Jorge Barroso Dias, Fabriziomaria Gobba, Swen Malte John, Tom Loney, Cristina Pinho, Ana Rodrigues, Claudine Strehl, Thomas Tenkate, Marc Wittlich, Alberto Modenese

**Affiliations:** ^1^CHRC, NOVA Medical School, Faculdade de Ciências Médicas, NMS, FCM, Universidade NOVAde Lisboa, Lisboa, Portugal; ^2^Institute of Public Health, College of Medicine and Health Sciences, United Arab Emirates University, Al Ain, United Arab Emirates; ^3^Institute for Interdisciplinary Dermatological Prevention and Rehabilitation (iDerm) at Osnabrück University, Osnabrück, Germany; ^4^Department of Dermatology, Environmental Medicine and Health Theory, Institute for Health Research and Education (IGB), Faculty of Human Sciences, Osnabrück University, Osnabrück, Germany; ^5^UNIDEMI, NOVA School of Science and Technology, NOVA University Lisbon, Caparica, Portugal; ^6^Instituto Português do Mar e da Atmosfera, Atmosfera, Portugal; ^7^Institute for Occupational Safety and Health of the German Social Accidents Insurance (IFA), Saint Augustin, Germany; ^8^Departamento de Saúde, Higiene e Segurança, Câmara Municipal de Lisboa, Lisboa, Portugal; ^9^Portuguese Society of Occupational Medicine, Working Committee "Work at Open Air", Lisboa, Portugal; ^10^Department of Biomedical, Metabolic and Neural Sciences, University of Modena and Reggio Emilia, Modena, Italy; ^11^College of Medicine, Mohammed Bin Rashid University of Medicine and Health Sciences, Dubai, United Arab Emirates; ^12^School of Occupational and Public Health, Toronto Metropolitan University, Toronto, ON, Canada

**Keywords:** outdoor workers, occupational health, solar ultraviolet radiation (UVR), digital public health, non-melanoma skin cancer

## Abstract

**Introduction:**

The rising incidence of skin cancer over the years has made it a significant public and occupational health issue. However, skin cancer is highly preventable, mainly through reduced exposure to solar ultraviolet radiation (UVR), which can be achieved by a variety of individual and collective protective measures and interventions. The relative risk associated with different patterns of exposure to solar UVR differs for the subtypes of keratinocyte cancers (KC). Specifically, whether the exposure is intermittent or continuous, and occurs in an occupational or leisure/recreational setting. The main aim of the study using this protocol is to contribute to raising public and policy awareness on solar UVR-inflicted occupational skin cancers in Lisbon. This will be achieved by performing direct measurements of the solar UVR dose received by outdoor workers using a digital platform. Results will likely contribute to further understanding the risk estimates for keratinocyte cancer estimations in this population.

**Methods:**

A prospective observational study will be conducted in Lisbon, Portugal. Personal electronic dosimeters (GENESIS-UV system) integrated with a digital platform will be used to assess occupational solar UVR doses of gardeners, masons, and gravediggers of the municipality of Lisbon. Two hundred and ten outdoor workers will be selected to wear the dosimeter for 1 month each, between April and October during their daily working hours. A digital web-based platform that offers private access to information through dashboard visualization will provide information for the outdoor workers and facilitate communication with the participants.

**Discussion:**

The expected results of the overall proposal comprise the occupational solar UVR doses, expressed in standard erythemal dose (SEDs) per day of outdoor work for 7 months. Study data will provide outdoor workers with information on their personal solar UVR exposure during their working hours and an estimate of their risk of developing skin cancer. It is expected that the occupational solar UVR doses of the outdoor workers in Portugal will be above the threshold of 1 to 1.33 SED/day, due to the latitude of Lisbon and the nature of the occupations. The results prospectively should flow into the design of adequate prevention campaigns for skin cancer in outdoor workers.

## 1. Introduction

The rising incidence of skin cancer over the years has made it a significant public and occupational health issue (1, 2). In 2017, there were approximately 7.7 million new cases of keratinocyte carcinomas (KC) worldwide, [that belong to the group of non-melanoma skin cancer (NMSC)], of which 5.9 million were basal cell carcinoma (BCC) and 1.8 million were cutaneous squamous cell carcinoma (cSCC) (3). The Global Cancer Observatory (GLOBOCAN) of the International Agency for Research Cancer (IARC) estimates an incidence of 1.2 million NMSC (excluding BCC) and 63,731 deaths due to this type of cancer in 2020 (4). These estimates do not reflect the circumstances during the severe acute respiratory syndrome coronavirus type 2 (SARS-CoV-2) pandemic and are based on extrapolations of cancer data from the years before (4). The incidence of KC varies widely by geographic location and ethnic skin type (5, 6) but is the most common cancer in fair-skinned populations across the world (7), that are predominantly of European ancestry. The relative risk associated with different patterns of exposure to solar UVR differs for the subtypes of KC. BCC is most strongly related to a history of sunburns and measures of intermittent exposure to the sun, such as sunbathing and beach vacations, accounting for approximately 50–90% of BCC. In contrast, cSCC is most strongly associated with continuous patterns of sun exposure, including occupational exposures (8). In Europe, skin diseases (i.e., contact dermatitis, contact urticaria, and skin cancer) constitute up to 40% of all notified occupational diseases (9). The new 11th International Classification of Diseases (ICD) revision (ICD-11) now includes cSCC and BCC in two separate categories, respectively with codes 2C31 and 2C32 (10). Moreover, it provides the possibility to add further codes to indicate occupation as the primary factor for the disease, or as a cofactor, or to code the tumors as not occupation-related.

Ultraviolet (UV) radiation (UVR) is part of the spectrum of electromagnetic radiation emitted by the sun, and it is arbitrarily divided into three bands of different wavelengths: (i) UVA 400–315 nm; (ii) UVB 315–280 nm; and (iii) UVC 280–100 nm. Shorter wavelength cause more DNA damage to the skin compared to longer wavelengths. Solar UVR reaching the Earth’s surface only contains UVA and UVB bands (11) and these are the two types of UVR primarily responsible for skin malignancies (12), including KC. In 2012, the IARC confirmed the classification of solar UVR as Group 1 carcinogen, meaning that there exists sufficient evidence about its carcinogenicity to humans (13).

The exposure level is a determinant factor for the development of skin cancer (14). The dosimetry of UVR exposure of the eye and skin requires the use of several radiometric quantities and units, the radiant exposure (J/m^−2^) is the accumulated radiant energy per unit area in joules per square meter (Jm^−2^) (15). The International Commission on Non-Ionizing Radiation Protection (ICNIRP) describes the acceptable daily exposure at each UV wavelength as being equivalent to approximately 1.0 to 1.33 Standard Erythema Dose (SED) (16). This “standardized dose” assumes that 1 SED equals 100 Jm^−2^ of erythemal effective UVR exposure (16). Another related quantifying measure used in public health is the UV Index, which indicates the risk of sunburn under given meteorological conditions. A UV Index of 1.0 corresponds to 0.9 SED per hour (15). Considering chronic solar UVR exposure at work, one of the latest available definitions of workers at risk for adverse skin effects has been applied in Germany. The German criteria for the recognition of occupational skin cancers, is the condition of working more than 1 h outdoors between 11 a.m. and 4 p.m. for more than 50 days during the period April–September in the northern hemisphere (17).

For outdoor workers, solar UVR is the most relevant occupational carcinogenic exposure (14). Outdoor workers are often exposed to higher doses than the theoretical minimum exposure level (1 to 1.33 SED/day). A systematic review serving the WHO/ILO joint project for the estimation of the global burden of diseases related to occupational solar UVR exposure has identified the threshold of occupational exposure to solar UVR as ⫺0.33 SED/day (12), or through a proxy of occupation, occupational group, job task or other variables. The meta-analysis conducted including an overall of 457,360 participants (melanoma and KC) estimated a significant increased risk of 60% in the incidence of NMSC for outdoor workers (RR: 1.60; 95% CI: 1.21–2.11), without any significant differences between sex and geographical region. The increased risk of basal cell carcinoma (RR: 1.50; 95% CI: 1.10–2.04) was lower than the increased risk for squamous cell carcinoma (RR: 2.42; 95% CI: 1.66–3.53) (18).

A variety of individual and collective protective measures can be used to reduce solar exposures which will assist in the prevention of skin cancer. Behavior change interventions on the skin cancer continuum (prevention, detection, diagnosis, treatment, and survivorship) should be designed under a multiple-component and intensive strategy for action to be successful (19, 20). Health education has been the target of these programs but there are other possible simple measures to implement that may increase the efficiency of behavior change (21). Clear risk communication is vital for enhancing hazard awareness and engagement with behavior change and the adoption of good practices at work (21). Primary prevention of occupational risks at the workplace also requires macro-support and reinforcement from governmental and institutional preventive actions and policies, norms, and guidelines (9). Sun-safety information and training of the workers, roofing of outdoor workplaces, use of panels and glasses to reduce solar UVR, and organizational measures (work breaks, breaks in shaded places, breaks or inside work during the middle hours of the day) are examples of collective preventive measures. Examples individual measures include appropriate personal protective equipment (PPE) for the workers (sunglasses, broad-brimmed helmets and hats, long-sleeves shirts and UVR filtering clothes, and sunscreens) (1, 9).

Recently, the use of communication devices and their integration with digital public health suggests that mobile apps can be used to drive behavior change aiming to prevent diverse types of cancer, including skin cancer. A recent review identified seven mobile apps that can be used for skin cancer early identification (22), and there are also generic apps targeting sun safety, such as the Sun Smart app from the Council Victoria and the Victorian Government of Australia (23). Moreover, the WHO and the ILO, together with the World Meteorological Organization and the United Nations Environment Programme (UNEP), recently launched the SunSmart Global UV App in June 2022, which provides five-day UV and weather forecasts (24). Two other systematic reviews highlighted that skin cancer prevention apps need to be tailored to specific participant groups, and studies including personalized messages for outdoor workers have also found more effective results in sun safety protection (25, 26). Likewise, these digital platforms can play an important role in managing health risks by improving the deployment of workers in safer conditions. Properly validated digital health solutions (e.g., web-based or mobile apps) offer the opportunity for person-centered action for cancer prevention and treatment, although it is recognized that this is a growing area, and therefore, needs more attention (27). Tailored digital health measures according to each health profile and personalized nudging (e.g., reminders by messaging), are examples of effective solutions for long-term health promotion, where positive behavior change is key (28).

## 2. Methods and analysis

### 2.1. Design and setting

**Figure 1:**
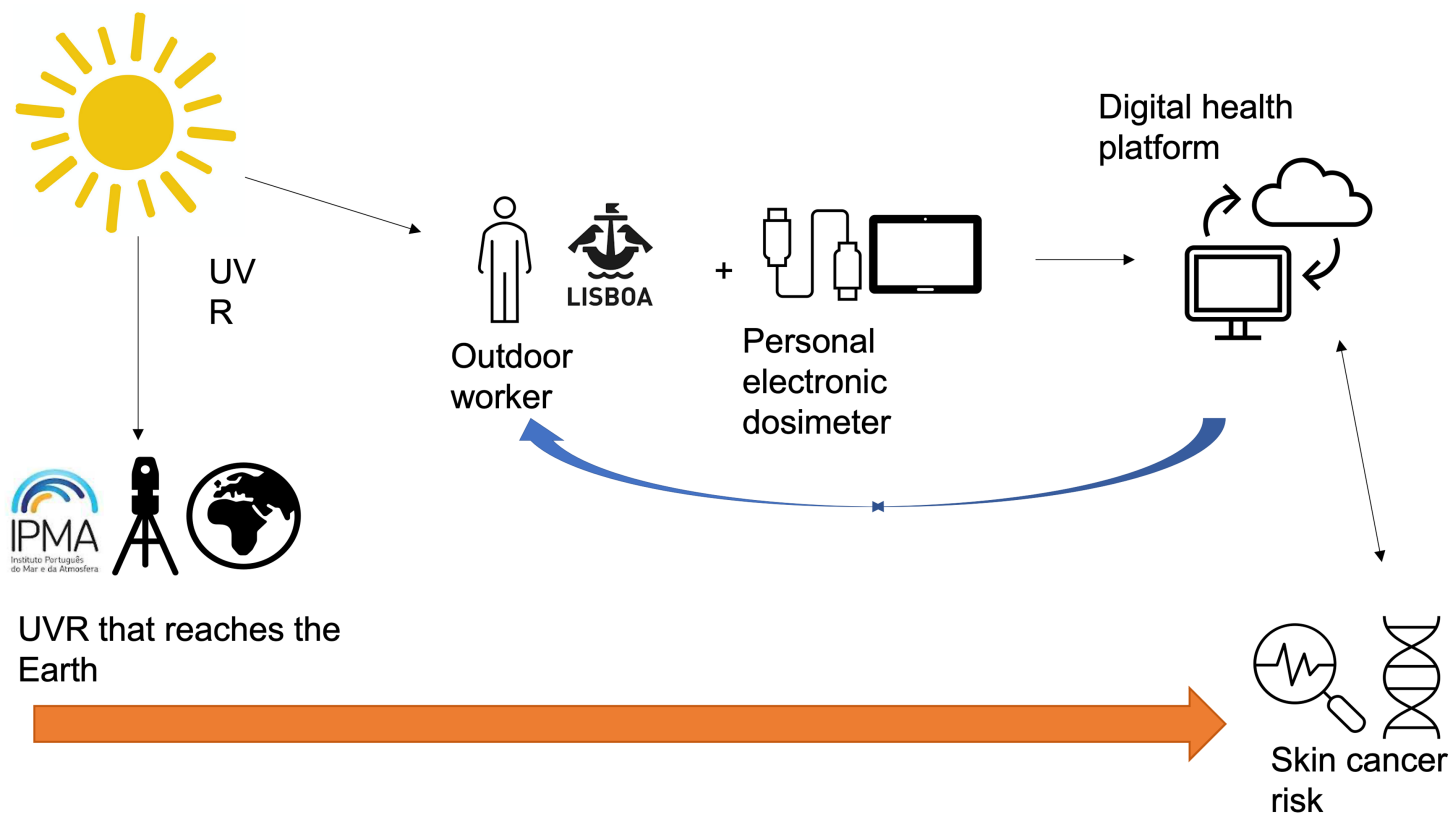
Conceptual map of the study design.

A prospective observational study will be conducted in Lisbon municipality area, Portugal between April to October 2023. The study will include an occupational solar UVR exposure evaluation of a sample of outdoor workers with personal electronic dosimeters, a specific training intervention for the prevention of skin cancer risk, and a screening through skin examination of the workers provided by expert dermatologists, to estimate their skin cancer risk. The target group of outdoor workers includes gardeners, masons, and gravediggers of the municipality of Lisbon (CML). Figure 1 presents the study’s conceptual design.

### 2.2. Participants and recruitment

The population of outdoor workers from the municipality of Lisbon comprises 174 gardeners, 27 masons, and 88 gravediggers. A sample of 30 outdoor workers (10 per occupation) will be invited to wear the dosimeter for 1 month. After 1 month, the dosimeters will be assigned to another sample of ten outdoor workers from the same occupation. For occupations with a smaller number of employees, the dosimeters will rotate among them during the 7 months, for example, each mason will likely wear the dosimeter for 3 months (not consecutively). All the outdoor workers from a specific occupation will be invited to participate in the study.

### 2.3. Description of the study procedures

#### 2.3.1. Data collection

Personal electronic dosimeters following the GENeration and Extraction System for Individual exposure to UV (GENESIS-UV) (29) will be used by outdoor workers from Lisbon municipality to collect the data and register it in a database. The electronic data logger included in the GENESIS-UV system is an X-2012-10 (Gigahertz, Turkenfeld, Germany) UV dosimeter, measuring UV irradiance separately in the UVA and UVB/C bands. Each worker will be instructed to wear the dosimeter on the upper left arm in a standard position for all working days, usually from 9.00 a.m. to 5.00 p.m. The dosimeter collects data continuously according to the measurement time entered in advance (daily working hours per occupation).

Every week on Friday, participants will be required to connect with a cable from the dosimeter to a tablet provided to the workers (with the support of researchers), as part of the GENESIS-UV system, to automatically upload the data collected during the week and charge the dosimeter. This task will be performed by a trained Health and Hygiene Technician to ensure compliance and best practice. Through the tablet connected to the internet, the measured anonymized personal UVR exposure data [uploaded and stored in an institutional safe server, in compliance with General Data Protection Regulation (GDPR)], will be processed and analyzed, according to the GENESIS-UV mathematical model for UVR exposure estimation. Uploaded data will be processed as daily and monthly averages (expressed in W/ m^2^) and cumulative solar UV erythemal doses (expressed in J/m^2^), which are then converted into SED (Standard Erythema Doses). In the end, dosimeters provide the “biologically weighted” SED. One SED corresponds to 100 J/m^2^ (normalized to 298 nanometers). The measurement campaign will last from April to October 2023.

#### 2.3.2. Training session

Before the provision of the dosimeters to the outdoor workers, an education training session will be organized in collaboration with the CML, which will be responsible for the outdoor workers’ coordination. The objective of this session will be to brief the outdoor workers about the objectives of the project and the necessary procedures needed from them, such as the correct use of the dosimeters, the risks of solar exposure, and protection measures. The dosimeters will be the responsibility of the direct supervisor of the teams of outdoor workers assigned to the research, which will be then supervised by the Health and Hygiene Technician who is part of the Health and Safety Department of the Occupational Health Services of CML. There will be 30 dosimeters available for teams of 10 masons, 10 gravediggers, and 10 gardeners. To increase compliance with the use of the dosimeters, each outdoor worker will use the dosimeter for 1 month, and then the dosimeters will rotate for other outdoor workers from the same occupation.

At the end of the seven-month data collection, the data from 70 months of measurement will be available for each of the three occupational group’s gardeners, masons, and gravediggers.

#### 2.3.3. Dermatological screening

Dermatologists will perform a consultation with 210 outdoor workers from CML. This activity will map the current acute and chronic aspects of the skin exposed to chronic occupational solar UVR and it will include the collection of information about the worker’s health status and the dermatological screening itself.

The objective examination using digital dermoscopy will include the screening for skin cancers and pre-neoplastic lesions, nevi count by segment as a marker of cumulative sun exposure, registration of clinical indices of cutaneous photoaging, and evaluation of markers of cumulative exposure to ultraviolet radiation by ultrasound and *in vivo* confocal microscopy of the skin.

#### 2.3.4. Development of the digital health platform

A digital platform for health monitoring has been implemented—METHIS (Multimorbidity Management Health Information System, a web-based platform that provides digital services for healthcare professionals and patients at distance). This digital platform will support the upload and transform the data, in its unstructured format, as well as integrated and aggregated, to create a digestible and visual form, promoting easy, fast and comprehensive access to useful information whenever needed (28). The Digital Portal was designed using Design Science Research Methodology and features offer private access to solar UVR exposure information through dashboards visualization to workers and safety officers or masons-supervisors.

The Digital Portal’s design and features were designed to allow its users to manage remotely the chronic diseases (and other conditions) under the Goal-Oriented Care logic, where communication between the healthcare professionals and each patient is promoted. This type of interventive research approach, based on accessing its daily performance, allows adapting this platform to outdoor workers and their occupational medicine team. Moreover, the system permits each worker to participate actively in the study (e.g., following closely the data collection process), which is essential for proper enrolment and engagement. During the study timeline, communication with the participants and mediators will be of major importance for engagement and compliance improvement.

#### 2.3.5. Collection of environmental data

Data from the weather stations nearby the study sites (provided by the Portuguese Institute for Sea and Atmosphere, IPMA) will be collected and correlated with the dose captured by the personal electronic dosimeters. Collected dose data will also be compared with model results from IFS (Integrated Forecasting System) provided by CAMS (Copernicus Atmosphere Monitoring Service) to evaluate their adequacy in human UV exposure monitoring, enabling further estimations of occupational exposure based on predictive models using environmental UVR data.

### 2.4. Statistical methods and data analysis

Descriptive statistics will be used to characterize our sample by the socio-demographic variables (sex, age, occupational group, personal and family history of skin cancer, number of years as outdoor workers, the average number of hours exposed to the sun per day, risk behavior at work and during leisure activities, adoption of protective measures at work and during leisure activities, history of sunburns, risk perception of UV radiation, and health literacy). After completing the data collection process, and the translation of the physical solar UVR measurements from J/m^2^ into SED, statistical analysis to describe the mean and standard error of the measurements will be performed. Data will be analyzed as pooled data from each occupation studied.

Statistical software for data science (STATA) statistical software will be used for all the mentioned analyses.

## 3. Discussion

The field of dermato-oncology will likely become more relevant in the coming years (30). It is to be expected that higher incidences of (occupational) skin cancer cases will lead to a precarious care situation, as medical experts (i.e., dermatologists) will be confronted with more patients that they need to treat (31). It is of high importance that strategies are developed to prevent new malignancies in people already diagnosed with (occupational) skin cancer. In this, actions must be taken in terms of primary, secondary, and tertiary prevention, with differing focuses according to the individual goal of the respective preventive stage. The severe under-reporting might be tackled also by rising awareness amongst the workers and their employers (32).

Recent research has shown that outdoor workers’ perceptions of skin cancer risk and attitudes to sun-protective measures need to be considered to design effective prevention strategies. Further, special requirements of outdoor workers need to be considered in the design of adequate preventive measures (33). As has been shown in Australia (i.e., “Slip! Slop! Slap! campaign”), sun safety behavior in the general population can be addressed by tailored interventional, and educative programs (34). The presented project also intends to contribute to furthering the evaluation of key features for designing adequate interventions in terms of sun-safe behavior in the vocational area, especially for outdoor workers. The access to real time data enables a faster and more accurate response to this problem.

Furthermore, it is important to raise the awareness of policymakers and other occupational health and safety prevention stakeholders, to have the occupational risk acknowledged and to improve the reporting of occupational skin cancers, currently largely unrecognized. Recent data shows an increase in the reporting also in Italy after these types of interventions (35).

If the methods succeed, the approach can be used to study new relations between exposure and effects, as there are other fields of interest for prevention purposes, e.g., targeting the exposure evaluation for the eyes to investigate ocular diseases such as cataracts, ocular melanoma, and macular degeneration. While for the skin there are emerging data suggesting that skin melanoma subtypes such as Lentigo Maligna Melanoma (LMM) (18) can be related to cumulative occupational solar UVR exposure. The recent WHO-ILO systematic review meta-analysis found an increased RR of 1.45 (CI 95% 1.08–1.94) in the studies specifically including LMM related to occupational solar UVR exposure (18). Finally, with the same approach, future studies may want to consider the beneficial effects of solar UVR exposure, as the minimum recommended exposure doses ensure healthy blood levels of vitamin D and subsequent bone health.

One of the limitations that we foresee is the worker’s compliance to wear the dosimeter during their working hours, including the turn-on or off in case they move to perform a task indoors. This level of uncertainty, however, needs to be tolerated and can be decreased by a thorough introduction of the workers to the approach during the training.

## 4. Ethics and dissemination

This study will be conducted under the principles of the 2013’s Declaration of Helsinki, therefore ethical approval is being sought by the Ethical Committee of NOVA Medical School|Faculdade de Ciências Médicas, Universidade Nova de Lisboa. Participation in the study will be voluntary, all the participants will sign an informed consent form before participation, and they will be free of renouncing their participation and the use of their data at any time. No investigations that can determine discomfort for the workers will be performed. The study will be conducted following the norms related to occupational health and safety at work, and it will contribute to the process of occupational risk evaluation and its prevention, which is mandatory for all companies, including the CML.

Data Protection and Impact Assessment (DPIA) process is also being conducted following the GDPR to improve the data protection risks possibly associated with the project and enable users will not to be at risk of any data violation. The DPIA is being developed in ARGOS which is a platform to simplify the process of creating and monitoring data management plans. The DPIA will be submitted to the site where the study will be conducted.

The dissemination plan of the current project proposal involves several communication strategies highlighted below that will be the responsibility of the task management committee. The project results will be submitted for publication results in international peer-reviewed open-access journals to allow unrestricted dissemination for the scientific community and the public in general. The research team also plans to present the findings at international and national conferences or any other important meetings from scientific organizations and groups, such as the Dermatology and Occupational Medicine Societies. Moreover, we aim to communicate the results of our study, specifically the results of occupational exposure to solar UVR in the national media channels, to raise awareness of this exposure and the use of personal protective equipment. This will be done in coordination with the respective academic press offices. A final seminar to present the results and discuss the implementation of policy measures.

## 5. Conclusion

One of the most important findings from the proposed project will be the expected combination of the results of the exposure assessment with the dermatological screening. Such data will result in the first comprehensive occupational UV-related skin cancer risk assessment for a group of outdoor workers, with an extremely relevant impact in terms of prevention, that should be further evaluated with follow-up investigations.

## Author contributions

MP conceptualized the study with the help of all authors. All authors have provided scientific content and helped to streamline the study protocol. All authors contributed to the article and approved the submitted version.

## Funding

This research is funded by Fundação para a Ciência e Tecnologia 2022.01888.PTDC.

## Conflict of interest

The authors declare that the research was conducted in the absence of any commercial or financial relationships that could be construed as a potential conflict of interest.

## Publisher’s note

All claims expressed in this article are solely those of the authors and do not necessarily represent those of their affiliated organizations, or those of the publisher, the editors and the reviewers. Any product that may be evaluated in this article, or claim that may be made by its manufacturer, is not guaranteed or endorsed by the publisher.
